# Supplementary material for: Atypical thioredoxin Patrx2 enhances alginate production in mucoid Pseudomonasaeruginosa
Source: Redox Biol. 2026 Jan 6;90:104010. doi: 10.1016/j.redox.2026.104010 (PMC12830090; doi:10.1016/j.redox.2026.104010)
Supplement: Multimedia component 1 [file mmc1.docx]

**Atypical thioredoxin Patrx2 enhances alginate production in mucoid *Pseudomonas aeruginosa***

Marie M. Grandjean, James N. Sturgis, Edwige B. Garcin, Moly Ba, Olivier Bornet, Christophe Bordi, Latifa Elantak and Corinne Sebban-Kreuzer*

Aix-Marseille Univ., CNRS, LISM UMR7255, IMM FR3479, Laboratoire d'Ingénierie des Systèmes Macromoléculaires, Institut de Microbiologie de la Méditerranée, 31 Chemin Joseph Aiguier, 13009 Marseille, France

***Corresponding author:** [corinne.kreuzer@imm.cnrs.fr](mailto:corinne.kreuzer@imm.cnrs.fr)

**Running title:** Patrx2 enhances alginate production in mucoid *P. aeruginosa*


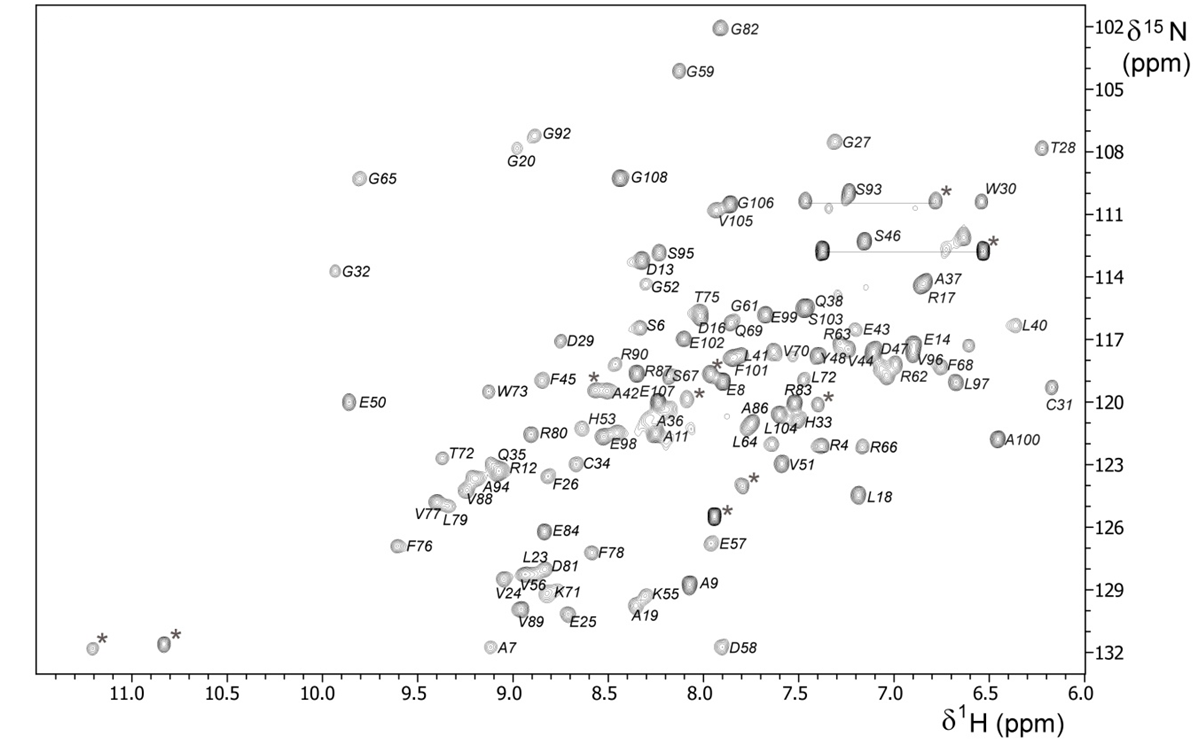


**Figures S1: ^1^H,^15^N-HSQC spectrum of oxidized Patrx2** in 150 mM NaCl, 10 mM KPO_4_ buffer pH8, 10 % D_2_O, at 300 K on a Bruker Avance III 600 MHz spectrometer. The backbone ^1^H,^15^N correlations are labeled according to the sequence. Side chain amine resonances are indicated with grey labels for Gln and Trp residues, and with grey star for Arg residues. Side chain resonances of Gln residues are connected by horizontal lines





**Figure S2: pKa determination of Patrx2 ionisable residues.** ^13^C NMR chemical shift pH titration curves for several C_β_ resonances. Apparent pKa values of histidine H33 and cysteine C34 in the reduced form of Patrx2 were determined. The pH-dependent chemical shift variation of the C_β_ carbons was measured, with the pKa defined as the pH at which 50 % of the chemical shift change is observed.


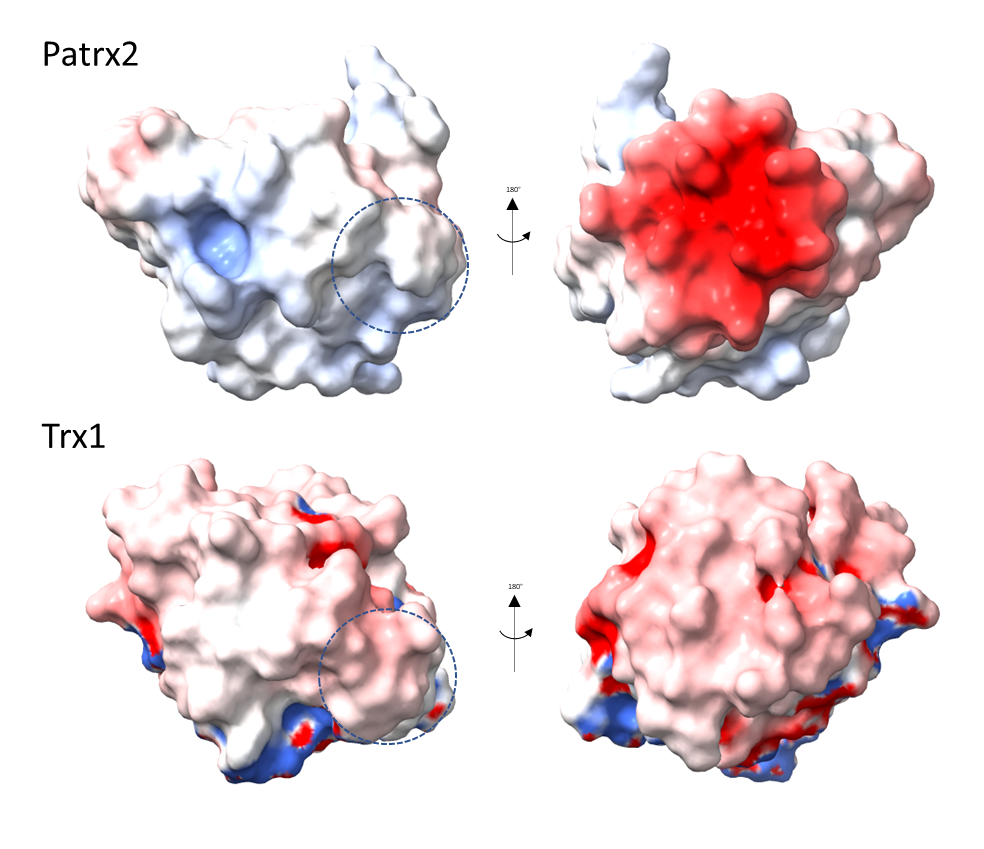


**Figure S3: Electrostatic surface potential comparison between Patrx2 and canonical E. coli Trx1.**

Electrostatic potential maps were generated using APBS after structure preparation with PDB2PQR, and visualized in ChimeraX using the command color electrostatic. Surface potentials are shown from two opposite orientations (180° rotation around the vertical axis) for each protein. Red and blue correspond to regions of negative and positive electrostatic potential, respectively, with the scale ranging from –10 kT/e (red) to +10 kT/e (blue). Compared to Trx1, Patrx2 exhibits a markedly different charge distribution: a more positively charged surface near the active site region (left panels, dashed circle), and an extended negatively charged region on the opposite face (right panels). These differences may reflect functional divergence between the two thioredoxins. Electrostatic surface potential representations of Patrx2 and E. coli Trx1 in two similar orientations; the CXXC site is encircled by a dashed circle for both proteins.


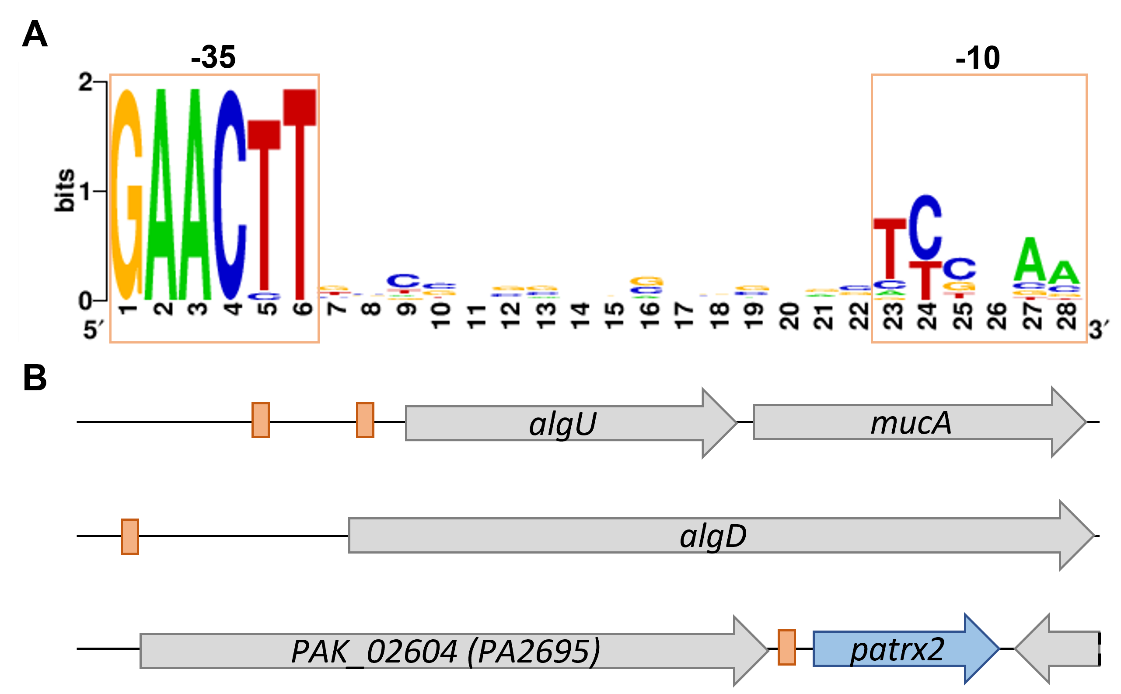


**Figure S4: Identification of a putative AlgU binding site upstream of *patrx2*. A.** Sequence logo of the AlgU binding motif defined using MEME based on published AlgU-dependent promoter. Conserved -35 (left box) and -10 (right box) elements are highlighted in green. **B.** Genomic context of known AlgU-regulated genes (*algU*, *algD*) and the *patrx2* locus in *P. aeruginosa* PAK. Green boxes indicate predicted AlgU-binding sites identified using FIMO. For *algU*, binding sites were detected 58 bp (p = 2.02×10⁻⁵, q = 0.0252) and 242 bp (p = 2.67×10⁻⁷, q = 0.001) upstream of the start codon. The *algD* site is located 372 bp upstream (p = 1.74×10⁻⁵, q = 0.0252), and the *patrx2* site lies 33 bp upstream (p = 3.64×10⁻⁵, q = 0.0341).


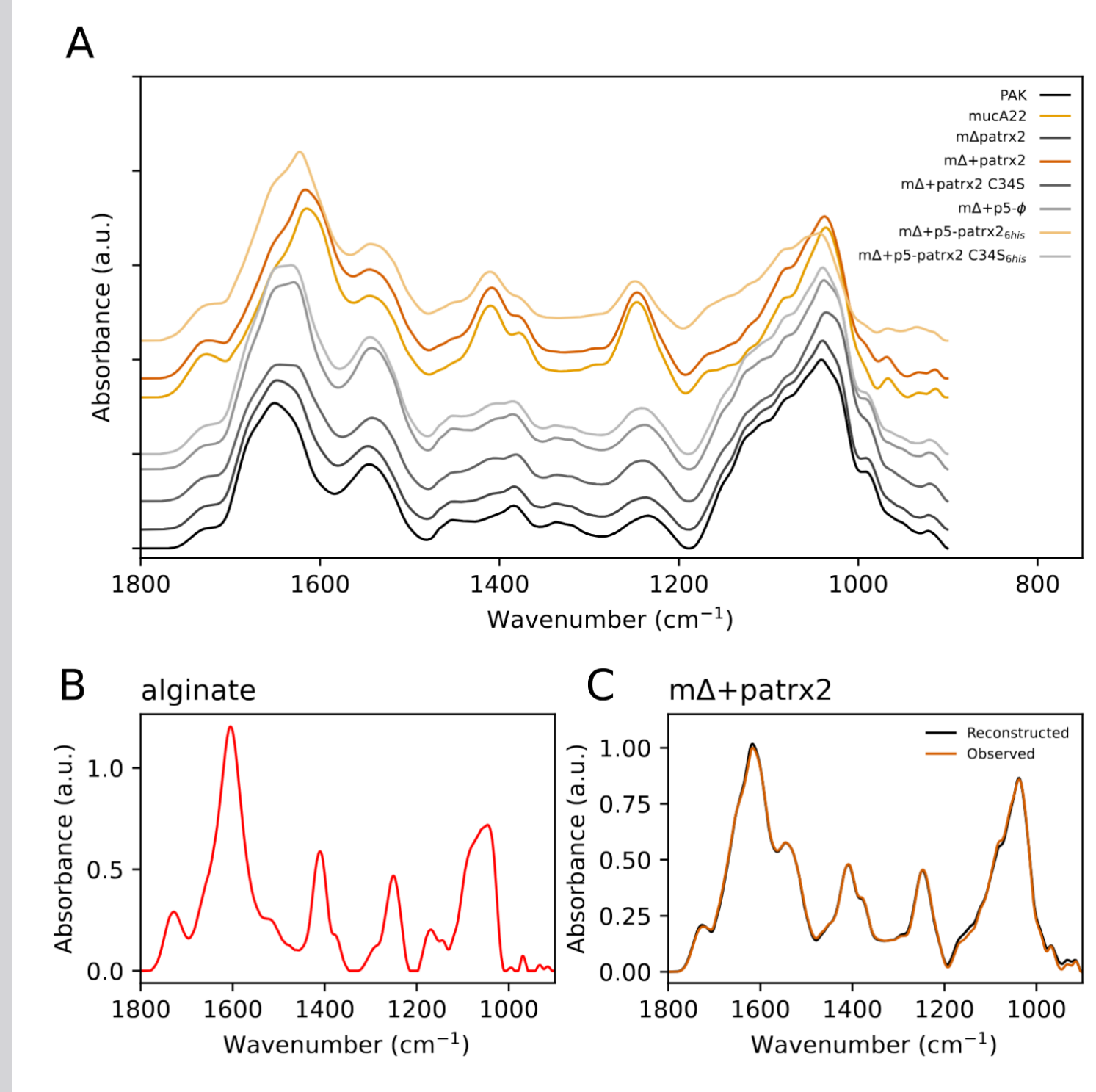


**Figure S5. FTIR-based analysis of biofilm matrix composition. A.** FTIR spectra of biofilm extracellular matrix, extracted by two rounds of isopropanol precipitation and resuspended in water, from eight *P. aeruginosa* strains (PAK, PAK*mucA22* (*mucA22*), PAK*mucA22∆patrx2* (*m∆patrx2*), PAK*mucA22∆patrx2::attBpatrx2* (*m∆+patrx2*), PAK*mucA22patrx2C34S* (*m:patrx2C34S*)*,* PAK*mucA22∆patrx2+*pBBRMCS5-Ø (*m∆+*p5-*Ø*)*,* PAK*mucA22∆patrx2+*pBBRMCS5*-patrx2_6His_* (*m∆+*p5*-patrx2_6His_*)*,* PAK*mucA22∆patrx2+*pBBRMCS5-*patrx2C34S_6His_* (*m∆+*p5*-patrx2C34S_6His_*)). Residual water contributions were removed, baselines corrected, and spectra were normalized to the C–O modes (1200–950 cm⁻¹). Spectra have been shifted vertically for visibility. **B.** FTIR spectrum of alginate contribution identified by NMF. **C.** FTIR spectrum of the *mucA22 Δpatrx2* strain complemented with chromosomal *patrx2* (orange), overlaid with its NMF-based theoretical reconstruction (black), illustrating the quality of the six-component decomposition. The component corresponding to alginate was assigned based on its characteristic bands at 1606 and 1410 cm⁻¹ (carboxylate C=O stretching), 1732 and 1252 cm⁻¹ (O-acetyl ester vibrations), and its similarity to the commercial alginate reference.

**Table S1:** Bacterial strains, plasmids and oligonucleotides used in this study

| *E. coli* strains | Relevant characteristics | Reference |
| --- | --- | --- |
| DH5α | *fhuA2 lac(del)U169 phoA glnV44 Φ80' lacZ(del)M15 gyrA96 recA1 relA1 endA1 thi-1 hsdR17* | Lab collection |
| TG1 | K-12 *supE thi-1 Δ(lac-proAB) Δ(mcrB-hsdSM)5, (rK-mK-)* | Lab collection |
| CC118(λpir) | Host strain for pKNG101 replication, Δ(*ara-leu*) *araD* Δl*ac*X74 *galE* *galK phoA20 thi-1 rpsE rpoB* *argE*(Am) *recA1* Rf^R^ (λpir) | Lab collection |
| Sm10 | *thi-1 thr leu tonA lacY supE recA::RP4-2-Tc::Mu (*Km*^R^) supE44* | Lab collection |
| *P.* *aeruginosa* strains | **Relevant characteristics** | **Reference** |
| PAK | Wild type | Lab collection |
| PAK*patrx2-6H* | PAK strain with a tag encoding 6 histidine residues fused to the 3' end of the *patrx2* gene. | This study |
| PAK*Δpatrx2* | PAK strain with deletion of the coding sequence of the *patrx2* gene | This study |
| PAK*Δpatrx2::attBpatrx2* | PAK*Δpatrx2* strain with a *plac-patrx2-STtag* fusion inserted at the *attB* site, (Tc^R^) | This study |
| PAK*patrx2C34S* | PAK strain carrying the *patrx2* gene with C34S substitution. | This study |
| PAK*p_patrx2_-lacZ* | PAK strain with deletion of rbs and the coding sequence of *patrx2* gene, substituted with the rbs and the *lacZ* gene sequence. | This study |
| PAK*mucA22* | PAK strain with a guanine deletion at position 425 in the *mucA* gene, resulting in a frameshift and the introduction of a stop codon at position 441. | This study |
| PAK*p_patrx2_-lacZmucA22* | PAK*p_patrx2_-lacZ* with *mucA22* mutation | This study |
| PAK*mucA22patrx2-6H* | PAK*patrx2-6H* with *mucA22* mutation | This study |
| PAK*mucA22Δpatrx2* | PAK*Δpatrx2* with *mucA22* mutation | This study |
| PAK *mucA22Δpatrx2::attBpatrx2* | PAK*Δpatrx2::attBpatrx2* with *mucA22* mutation | This study |
| PAK*mucA22patrx2C34S* | PAK*patrx2C34S* with *mucA22* mutation | This study |
| Plasmids | **Relevant characteristics** | **Reference** |
| pJF119EH-*patrx2-6H* | pJF119-EH vector carrying the *patrx2-6H* gene, using BamHI and EcoRI sites. Ap^R^ | Lab collection |
| pBT20 | oriR6K γ, *ptac*, Gm^R^, Ap^R^ | Lab collection |
| pRK2013 | *Tra+ Mob+* Km^R^ *;* helper plasmid for triparental mating | Lab collection |
| pKNG101 | Suicide vector in *P. aeruginosa*, *sacB^+^*, Sm^R^ | Lab collection |
| miniCTX-*lacZ* | *lacZ^+^* ; self-proficient integration vector with *tet*, V-FRT-attPMCS, *ori*, *int*, and *oriT*, Tc^R^ | Lab collection |
| pBBR1MCS4 | Broad host range plasmid, Ap^R^ | Lab collection |
| pBBR1MCS4-patrx2 | pBBR1MCS4 carrying the *patrx2* gene, Ap^R^ | This study |
| pBBR1MCS5 | Broad host range plasmid, Gm^R^ | Lab collection |
| pBBR1MCS5-*patrx2-6H* | pBBR1MCS5 carrying the *patrx2-6H* gene, Gm^R^ | This study |
| pBBR1MCS5-*patrx2C34S-6H* | pBBR1MCS5 carrying the *patrx2C34S-6H* gene, Gm^R^ | This study |
| pKNG101-*p_patrx2_-lacZ* | Suicide vector for rbs and the coding sequence of *patrx2* gene, substituted with the rbs and the *lacZ* gene sequence by allellic replacement, Sm^R^ | This study |
| pKNG101-Δ*patrx2* | Suicide vector for *patrx2* deletion by allellic replacement, Sm^R^ | This study |
| pKNG101-*mucA22* | Suicide vector for *mucA*_Guanine 425_ deletion by allellic replacement, Sm^R^ | This study |
| pKNG101-*patrx2-6H* | Suicide vector for *patrx2-* 6his tag insertion by allellic replacement, Sm^R^ | This study |
| pKNG101-*patrx2-C34S* | Suicide vector for *patrx2-* C34S subtitution by allellic replacement, Sm^R^ | This study |
| miniCTX *plac-patrx2-ST* | *plac-patrx2-STtag* gene fusion inserted into miniCTX-*lacZ*, Tc^R^ | This study |
|  |  |  |
| Oligonucleotides | **Sequence (5’→3’)** | **Target** |
| *pKNG101-p_patrx2_-lacZ* |  |  |
| *patrx2-lacZ*-1 | ccctgcaggtcgacggatccCATGTATTTCGGCATCGCCC | PAK genome |
| *patrx2-lacZ*-2 | GATAAAGAAAGACAGGACGGCAGACTCCCGCG | PAK genome |
| *patrx2-lacZ*-3 | CCGTCCTGTCTTTCTTTATCACACAGGAAACAG | miniCTX-*lacZ* |
| *patrx2-lacZ*-4 | GGCTCTCGAAttatttttgacaccagaccaactgg | miniCTX-*lacZ* |
| *patrx2-lacZ*-5 | tcaaaaataaTTCGAGAGCCTGGTCGGCG | PAK genome |
| *patrx2-lacZ*-6 | cttatggtacccggggatccCCAACTGACCATGAGCGTGC | PAK genome |
| *pKNG101-Δpatrx2* |  |  |
| Δ*patrx2*-1 | CGGGATCCTTCAACGCCGGCTGGTATGC | PAK genome |
| Δ*patrx2*-2 | CCGGGTCCTCACATACGCTGCTTCTCCATGAGC | PAK genome |
| Δ*patrx2*-3 | AGCAGCGTATGTGAGGACCCGGCCCGCGGGC | PAK genome |
| Δ*patrx2*-4 | GGACTAGTCCATGAGCGTGCTCATGACC | PAK genome |
| *pKNG101-mucA22* |  |  |
| *mucA22*-1 | ccctgcaggtcgacggatcccaaggaccactgccggg | PAK genome |
| *mucA22*-2 | ggctacagcgaagagcaggggcgcc | PAK genome |
| *mucA22*-3 | gttggtgatcacctgcggcgcccctgctcttc | PAK genome |
| *mucA22*-4 | CTTATGGTACCCGGGGATCCaagcaatcgacaaagctctgcagcc | PAK genome |
| *pKNG101-patrx2-6H* |  |  |
| *patrx2-6H*-1 | GATTACGCGTTAACCCGGGCCCGCCACCGGCAAGACATTGCC | PAK genome |
| *patrx2-6H*-2 | CTAGTGGTGATGGTGATGATGGCCCTCGCCGACCAGGCTCTC | PAK genome |
| *patrx2-6H*-3 | CACCATCACCACTAGGGACCCGGCCCGCGGGC | PAK genome |
| *patrx2-6H*-4 | GGACTATAGACTATACTAGTGCTCATGACCCCCGACAAGG | PAK genome |
| *pKNG101-patrx2-C34S* |  |  |
| patrx2-C34S-1 | CGGGATCCTTCAACGCCGGCTGGTATGC | PAK genome |
| patrx2-C34S-2 | GCGGCCTGACTGTGGCCGCACCAG | PAK genome |
| patrx2-C34S-3 | GGCCACAGTCAGGCCGCCCAGCCG | PAK genome |
| patrx2-C34S-4 | GGACTATAGACTATACTAGTGCTCATGACCCCCGACAAGG | PAK genome |
| pBBRMCS-*patrx2* |  |  |
| *bbr-patrx2-1* | CCCTCGAGAGGAGGATATACCATGAA | *pJF119EH-patrx2-6H* |
| *bbr-patrx2-2* | GCTCTAGATCATTTTTCGAACTGCGGG | *pJF119EH-patrx2-6H* |
| *miniCTX plac-patrx2-ST* |  |  |
| *plac-patrx2-ST*-1 | CGGAATTCGAGCGCAACGCAATTAATG | pBBRMCS4-*patrx2* |
| *plac-patrx2-ST*-2 | CGAGCTCTCATTTTTCGAACTGCGGGTGGCTCCAGCCCTCGCCGACCAGGCTCTCG | pBBRMCS4-*patrx2* |

^*^Sm^R^, *aadA*, encoding aminoglycoside adenyltransferase for streptomycin resistance; Ap^R^, *bla*, encoding β-lactamase for ampicillin resistance; Gm^R^ *aacC1*, encoding aminoglycoside 3-N-acetyltransferase for gentamicin resistance; Tc^R^ *tetA*, encoding a tetracycline efflux pump for tetracycline resistance; Km^R^*, kan*, encoding aminoglycoside phosphotransferase for kanamycin resistance.

**Table S2: NMR and refinement statistics for Patrx2 structures**. Structural statistics and restraint violations of the 20 selected structures representative of Patrx2 in solution at pH 7.4 and 300K.

| **NMR distance and dihedral constraints** Distance constraints | | | |  |  |
| --- | --- | --- | --- | --- | --- |
|  | Effective distance restraints | | | | 2408 |
|  |  | Short range | (\|i-j\| ≤ 1) | | 1140 |
|  |  | Medium range | (1 < \|i-j\| < 5) | | 419 |
|  |  | Long range | (\|i-j\| ≥ 5) | | 849 |
|  | Average number of restraints per residue | | | | 23 |
|  | H-bond restraints | |  | | 63 |
|  | Dihedral angle restraints from TALOS | | | | 150 |
| **Structures statistics**  Restraint violations | | |  | |  |
|  | Distance >0,5Å | |  | | 1 |
|  | Dihedral >5° | |  | | 5 |
| Energies (kcal/mol) | | |  | |  |
|  | E_total_ | |  | | -3533 |
|  | E_bond_ | |  | | 60 |
|  | E_angle_ | |  | | 327 |
|  | E_dihedral_ | |  | | 1009 |
|  | E_VdW_ | |  | | -857 |
|  | E_electric_ | |  | | -6446 |
| Average ensemble RMSD (Å) | | | | |  |
|  | Backbone | |  | | 1.00±0.24 |
|  | Heavy atoms | |  | | 1.59±0.22 |
| Deviations from idealized geometry | | | | |  |
|  | bonds (Å) | |  | | 0.013 |
|  | angles (°) | |  | | 1.9 |
| Ramachandran plot (%)* | | | | |  |
|  | Most favorable region | | | | 87.9 |
|  | Additionally allowed region | | | | 12.0 |
|  | Generously allowed region | | | | 0.1 |
|  | Disallowed region | |  | | 0.0 |

* calculated using PROCHECK v. 3.5.4
